# V-shaped capsular plication for treating cam-type femoroacetabular impingement with hip instability in borderline dysplastic hips: a retrospective cohort study

**DOI:** 10.3389/fsurg.2026.1817849

**Published:** 2026-06-08

**Authors:** Yang Lv, Ningjing Zeng, Da Guo, Peng Yang, Hongliang Liu, Guangxi Chen, Xing Li, Dingkun Lin, Weiming Yang

**Affiliations:** 1 Department of Orthopedics, The Second Affiliated Hospital of Guangzhou University of Chinese Medicine, Guangzhou, Guangdong, China; 2 The Second Clinical Medical College, Guangzhou University of Chinese Medicine, Guangzhou, Guangdong, China

**Keywords:** borderline developmental dysplasia of the hip, capsular plication, femoroacetabular impingement, hip arthroscopy, instability

## Abstract

**Introduction:**

Purpose Patients with cam-type femoroacetabular impingement (FAI) frequently present with concomitant borderline developmental dysplasia of the hip (BDDH), a critical instability risk factor. Capsular plication may contribute to improved stability through the screw-home mechanism. This study evaluates the clinical outcomes of arthroscopic T-capsulotomy combined with V-shaped capsular plication for managing cam-type FAI in BDDH-associated instability and confirms its short-term efficacy.

**Methods:**

This retrospective cohort study included 15 patients diagnosed with symptomatic cam-type FAI and concomitant BDDH (LCEA: 20°-25°) between July 2019 and July 2023. All patients underwent arthroscopic labral repair, femoroacetabular osteoplasty, and T-capsulotomy with V-shaped capsular plication. Clinical outcomes were assessed using the Visual Analog Scale (VAS) for pain, Modified Harris Hip Score (mHHS), Hip Outcome Score-Activities of Daily Living (HOS-ADL), and Hip Outcome Score Sport-Specific Subscale (HOS-SSS) preoperatively and postoperatively. Statistical analysis employed paired t-tests for intra-group comparisons.

**Results:**

All 15 patients completed follow-up with a minimum duration of 12 months (mean 18.2 months, range 12–24). No major complications occurred. Significant improvements were observed in all outcome measures at final follow-up: mHHS improved from 57.33 ± 5.34 to 86.07 ± 2.67 (*P* < 0.01); VAS decreased from 5.73 ± 0.57 to 1.07 ± 0.57 (*P* < 0.001); HOS-ADL improved from 50.09 ± 5.02 to 89.31 ± 2.43; HOS-SSS improved from 41.66 ± 5.08 to 85.92 ± 4.70 (both *P* < 0.01).

**Conclusions:**

The described arthroscopic technique, adapting principles from pediatric DDH surgery, appears to be a promising approach for addressing cam-type FAI in BDDH patients with instability risk factors, demonstrating short-term clinical outcomes. But, further studies with larger cohorts and longer follow-up are warranted.

## 1 Introduction

A comprehensive understanding of hip joint stabilizers is essential in hip arthroscopy to effectively manage symptoms of instability and minimize iatrogenic complications. Beyond inherent bony stability, both static and dynamic soft-tissue stabilizers are crucial for maintaining hip joint congruity throughout the full physiological and even supraphysiological range of motion. The zona orbicularis and anterior capsular ligaments contain leash-like fibers in a spiral formation that tighten in a screw-home mechanism, enhancing stability in terminal extension and external rotation (1). During hip flexion, these fibers unwind and relax, which may contribute to reduced capsular tension and potential instability, particularly in positions of extension where the screw-home mechanism is most critical. It is noted that while the posterior capsule is a primary stabilizer in flexion, anterior capsular redundancy or laxity—particularly in dysplastic hips—can contribute to instability in positions combining flexion with external rotation. From a biomechanical perspective, capsulotomy during hip arthroscopy could potentially compromise joint stability (2). Routine capsular repair, however, may counteract this effect, restoring capsular integrity (3). Additionally, in cases involving ligamentous laxity or microinstability, tightening the capsuloligamentous structures can further reinforce the screw-home mechanism and help re-establish stability.

This article proposes a method to enhance hip capsular stability through T-capsulotomy followed by V-shaped capsular plication under hip arthroscopy. The aim of this technique is to excise the redundant lateral inferior capsule and, through V-shaped plication, shift the entire capsule from an external superior to an internal inferior direction, thereby theoretically exerting an inferomedial force on the femoral head to address the inadequate bony containment in BDDH. This technique is inspired by the Salter-type capsulorrhaphy, which is traditionally used in pediatric patients with developmental dysplasia of the hip (DDH) to tighten the hip joint capsule (4). Our approach replicates this effect arthroscopically, tightening the capsule from top to bottom and inside to outside. This maneuver is intended to compress the femoral head medially and inferiorly, addressing the deficient acetabular coverage characteristic of BDDH.

By emphasizing the spiral orientation of the fibers in the iliofemoral ligament at the anterior capsule, this approach enhances the screw-home mechanism of the hip, increasing capsular tension during extension and thereby reducing potential anterior instability in this position. This article aims to provide a comprehensive guide to the arthroscopic capsular plication procedure.

## 2 Methods

### 2.1 Inclusion and exclusion criteria

This retrospective cohort study was approved by the Ethics Committee (ZF2021-030-01), and all patients provided signed informed consent.

The inclusion criteria were as follows: (1) patients diagnosed with symptomatic cam-type FAI, confirmed by clinical examination (impingement sign), plain radiographs (elevated alpha angle >55° on Dunn view), and magnetic resonance imaging (MRI) demonstrating associated acetabular labral tears; (2) those diagnosed with combined BDDH, defined as a lateral center-edge angle (LCEA) between 20° and 25°; (3) those who underwent hip arthroscopy including the described capsular plication technique; (4) those aged between 18 and 60 years.

Exclusion criteria: (1) a history of previous ipsilateral hip surgery; (2) a history of acetabular fracture, avascular necrosis, Legg-Calvé-Perthes disease, bone tumor, or lumbar radiculopathy; (3) poor compliance and inability to cooperate with the study; (4) osteoarthritis of the hip (Tonnis grade >1).

### 2.2 Patient demographics

This study initially included 15 patients diagnosed with cam-type FAI and BDDH between October 2019 and July 2022. Among them, 2 were male and 13 were female (average age, 38.11 ± 13.44 years; average body mass index, 21.73 ± 3.11 kg/m^2^). Preoperative assessment of instability and data collection are performed for all patients based on symptoms, joint laxity and imaging findings, including the femoral external rotation angle (as determined by CT) and the Beighton score. As there is currently no universally accepted scoring system for hip microinstability, the clinical diagnosis of instability is based on a comprehensive assessment of clinical examination (impingement signs, manual laxity testing), patient-reported symptoms of instability, and radiographic findings consistent with marginal dysplasia. Radiographic measurements (LCEA, alpha angle) were performed independently by two orthopedic surgeons. Interobserver reliability was assessed using intraclass correlation coefficients (ICC > 0.85). Furthermore, due to the lack of standardised preoperative oblique radiographs, the anterocentral edge angle (ACEA) was not systematically measured in this study. All patients completed the minimum 12-month follow-up. Table 1 summarizes the demographic characteristics, preoperative imaging evaluation data, and surgical treatments.

**Table 1:** Patient demographics, preoperative imaging, and instability-related variables.

Patient lD	Age (years)	Sex	Side	BMI (kg/m^2^)	Tonnis angle (°)	Alpha angle (°)	LCEA (°)	Femoral version	Beighton score	Additional surgical procedures	Labral repair, No.	Length of follow-up, months
1	48	Female	Left	25.9	8	52	22	15.3	3	Femoral osteoplasty	1	60
2	48	Female	Right	22.3	9	55	21	18.7	3	Femoral osteoplasty	3	60
3	39	Female	Left	16.4	8	57	23	22.4	2	Femoral osteoplasty	3	48
4	18	Female	Right	19.2	13	58	20	14.2	3	Femoral osteoplasty and Acetabuloplasty	4	36
5	19	Male	Right	21.2	8	57	20	19.5	4	Femoral osteoplasty	2	36
6	47	Female	Right	23.4	7	53	20	11.8	2	Femoral osteoplasty	4	36
7	31	Female	Left	22.1	12	58	21	16.7	4	Femoral osteoplasty and Acetabuloplasty	4	36
8	32	Female	Left	26.1	8	51	22	12.5	1	Femoral osteoplasty	4	36
9	51	Female	Right	22.4	7	53	20	20.1	2	Femoral osteoplasty	5	36
10	57	Female	Right	21.6	11	54	20	17.3	3	Femoral osteoplasty and Acetabuloplasty	4	36
11	22	Female	Left	19.5	8	55	22	24.2	3	Femoral osteoplasty	3	36
12	53	Female	Left	26.5	8	52	22	13.9	2	Femoral osteoplasty	3	36
13	18	Female	Right	16.4	8	53	21	18.2	4	Femoral osteoplasty	3	24
14	51	Female	Right	18.9	11	55	22	10.5	1	Femoral osteoplasty and Acetabuloplasty	3	24
15	38	Male	Right	24.1	8	62	20	12.4	1	Femoral osteoplasty	4	24

### 2.3 Surgical technique

The mean operative time was 98.4 ± 12.6 min. The described capsular plication technique required approximately 15–20 min additional time compared to standard capsular closure. The learning curve for this technique was estimated at 10–15 cases to achieve consistent suture configuration and capsular tensioning.

#### 2.3.1 Anesthesia and position

All surgeries were performed by the same surgical team. Patients were placed in the supine position on a traction table under general anesthesia.

#### 2.3.2 Patient preparation and hip traction with fluoroscopy

**Figure 1:**
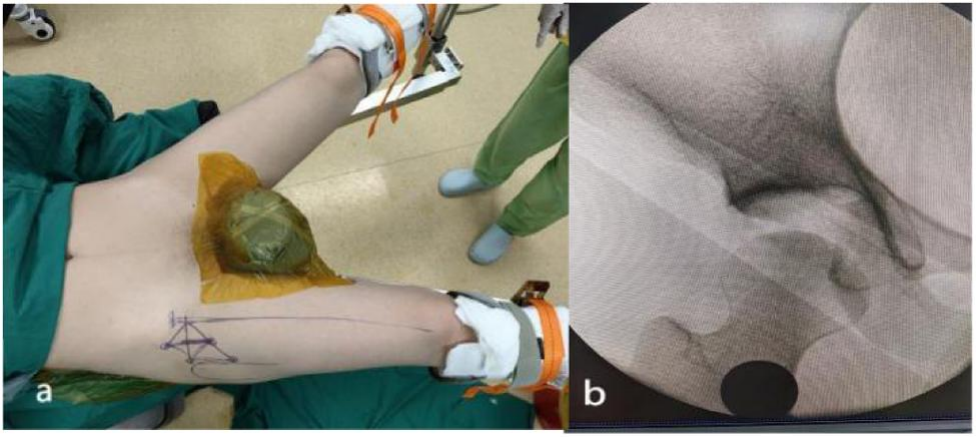
Demonstrates the positioning of the hip joint and imaging of the distracted hip joint space. **(a)** Shows the patient maintained in a supine position under traction. **(b)** Illustrates the hip joint space after adequate traction, with the joint space opening approximately 6–8 mm, revealing the “vacuum sign”.

A soft-padded perineal post was placed against the inner thigh of the affected side to protect against traction-related injury. Hip traction was guided using C-arm fluoroscopy. To obtain an accurate anteroposterior pelvic radiograph, the C-arm was adjusted as needed. The traction force on the affected hip was gradually increased to approximately 10–20 kg, widening the hip joint space by about 8–10 mm and creating a state of subluxation of the femoral head. This condition was typically confirmed by the appearance of an air sign within the joint, indicating disruption of the hip seal and successful joint distraction. At this stage, the positions of the greater trochanter, anterior superior iliac spine, and joint space were marked on the skin (Figure 1).

#### 2.3.3 Interportal capsulotomy and intra-articular diagnostic arthroscopy

**Figure 2:**
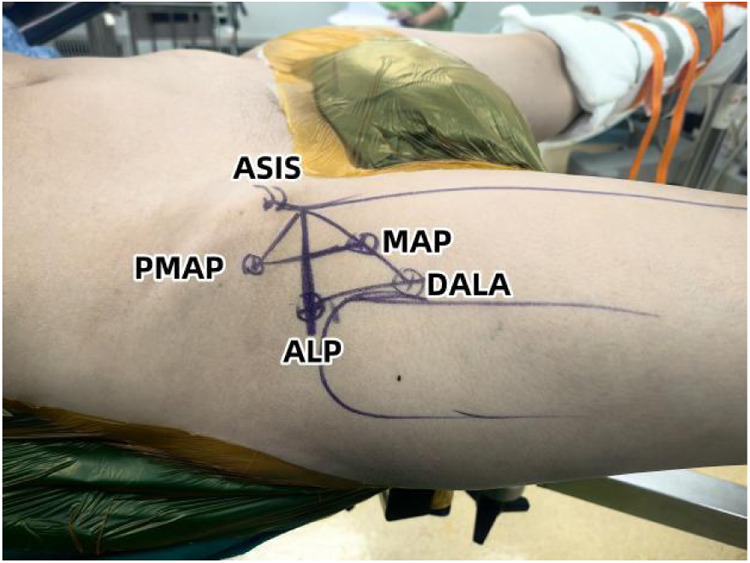
The position of the approach and hip arthroscopic operating tools required to be established during the hip arthroscopy procedure shown.

The anterolateral portal (ALP) and mid-anterior portal (MAP) were first established. A guide needle was reinserted through the MAP to ensure the approach avoided the acetabular labrum and femoral head, successfully accessing the hip joint space. Furthermore, the proximal mid-anterior portal (PMAP) was established in advance for capsule suturing (Figure 2). Using the over-the-wire technique, the hip arthroscope was advanced into the intracapsular space. The interportal capsulotomy was performed between the ALP and MAP using a low-temperature plasma knife (DePuy Orthopedics, Warsaw, IN, USA). The incision was made approximately 0.5 cm from the acetabular rim, with a length of 2–3 cm. Arthroscopic evaluation involved assessing labral tears, cartilage damage, and ligamentum teres tears. Procedures included femoral osteochondroplasty (cam resection), acetabular rim trimming, and labral refixation, as guided by imaging and clinical examination. In patients with evidence of pincer-type impingement or acetabular overcoverage, limited acetabular rim trimming was performed cautiously to avoid iatrogenic instability. The decision to perform acetabuloplasty was based on intraoperative findings and preoperative imaging, including crossover sign and acetabular version.

#### 2.3.4 T-capsulotomy and V-shaped capsular plication

First, based on the transverse (interportal) capsulotomy, the joint capsule was incised perpendicularly, parallel to the femoral neck, to create a T-shaped capsulotomy. This was extended distally until the circular fibers of the zona orbicularis were identified, typically at a length of 2–3 cm. Next, an arthroscopic shaver was used for limited debridement of the redundant inferior extracapsular tissue, preparing the area for subsequent capsular plication. This step significantly improved exposure of the femoral head-neck cam deformity, facilitating a more efficient and thorough osteochondroplasty.

A tissue grasper was then used to secure and advance the capsular flaps: Point A was located on the distal limb of the T-capsulotomy near the zona orbicularis, which was advanced superiorly to Point A' on the proximal native capsule near the acetabular rim. Similarly, Point B on the inferomedial flap was advanced superolaterally to Point B'. This maneuver tightened the hip capsule in superior-inferior and medial-lateral directions, theoretically compressing the femoral head inward and downward, facilitating a V-shaped reconfiguration.

**Figure 3:**
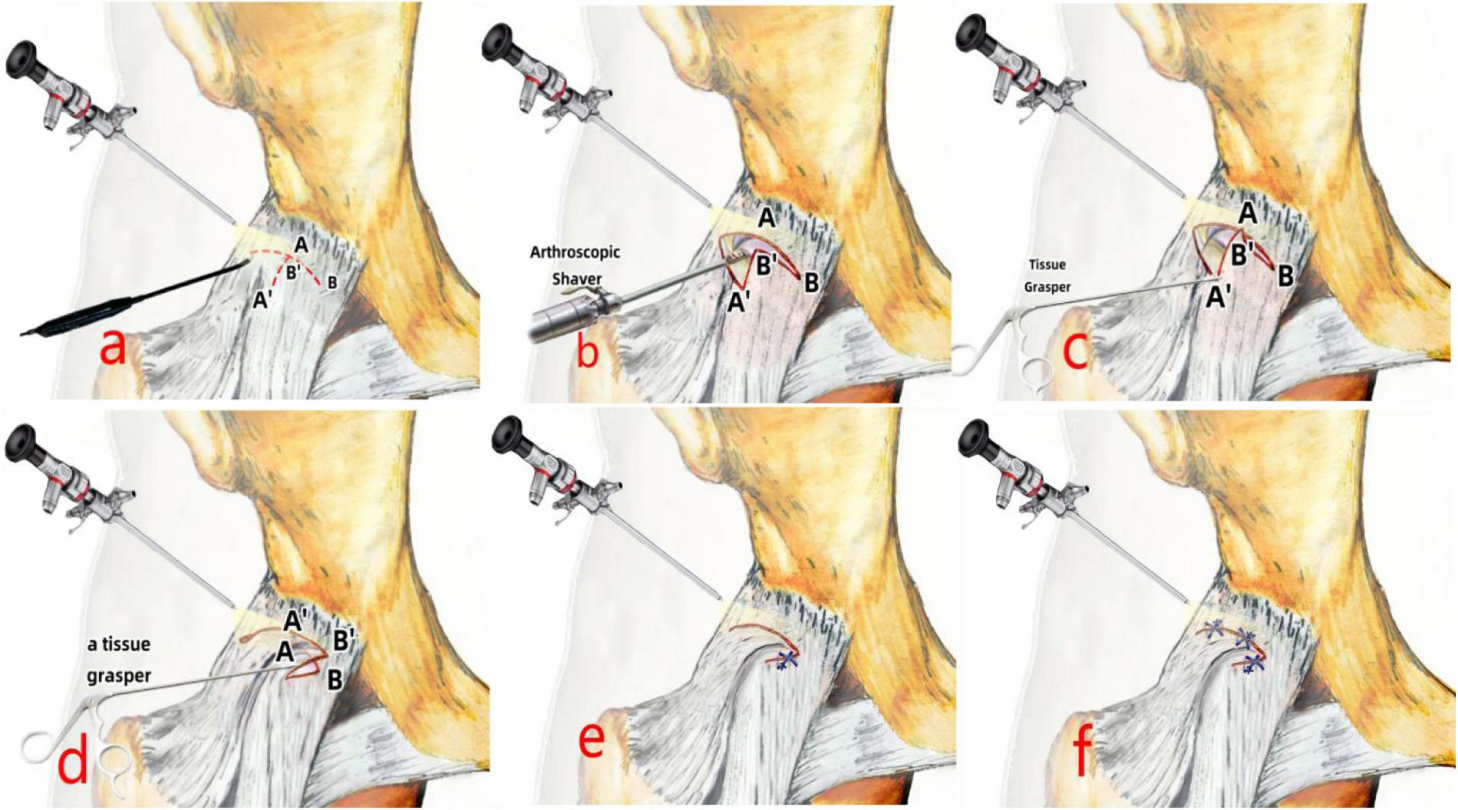
Arthroscopic surgical procedure for right hip capsular reconstruction. **(a)** Arthroscopic T-shaped capsulotomy performed using a radiofrequency ablation device; **(b)** Capsular débridement and contouring achieved with a motorized arthroscopic shaver; **(c)** Intraoperative demonstration of capsular advancement: A tissue grasper advances the capsular flap (point A') toward the native capsular margin (point A) to facilitate V-shaped plication; **(d)** Schematic illustration of V-shaped capsulorrhaphy for biomechanical restoration of the hip capsule; **(e)** Medial capsular closure through direct suture approximation of points B and B'; **(f)** Lateral capsular reinforcement using dual cross-stitch (X-configuration) sutures to complete the V-shaped capsular reconstruction.

**Figure 4:**
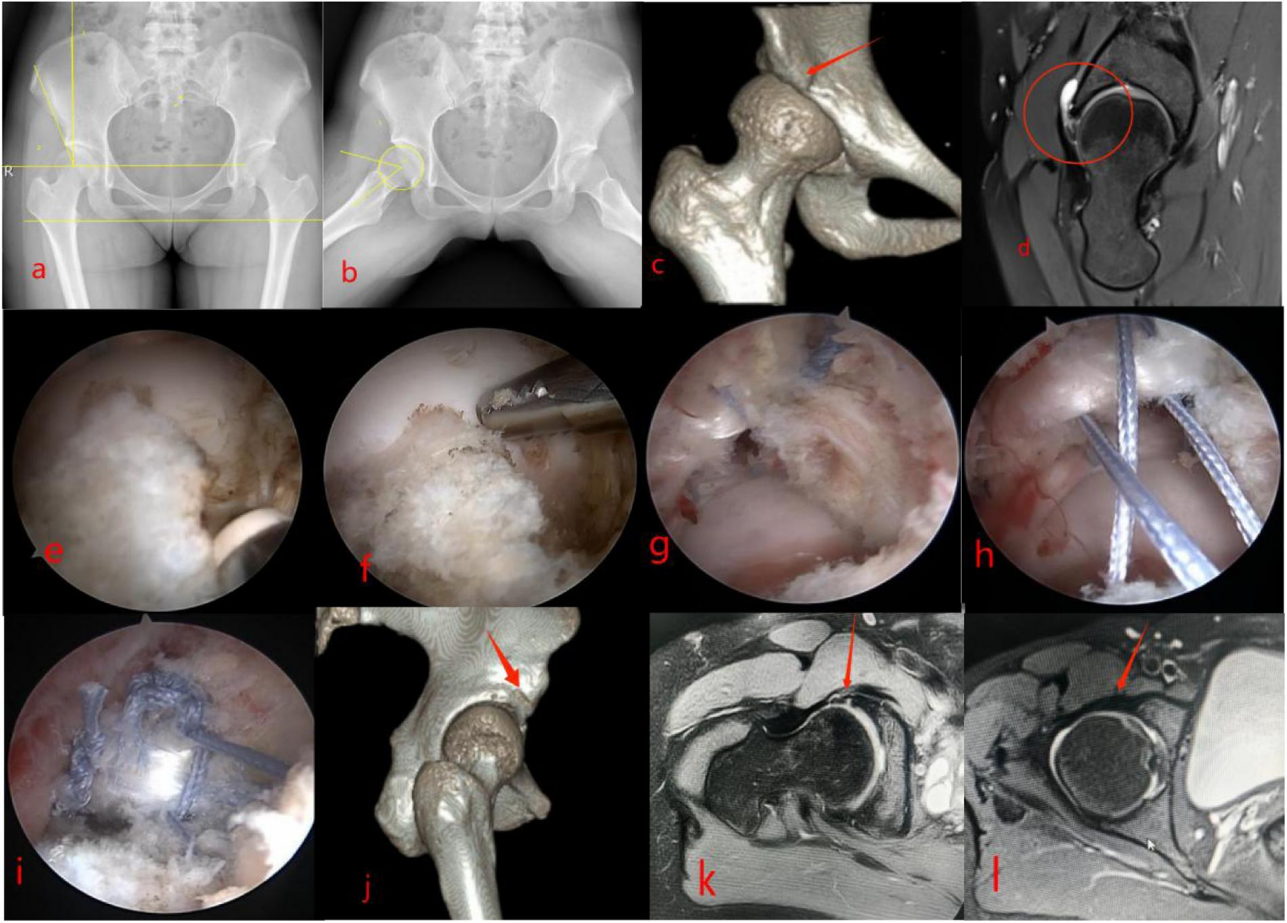
Representative case of arthroscopic labral repair, femoroacetabular osteoplasty, and capsular reconstruction in a 28-year-old female with symptomatic acetabular labral tear and borderline hip dysplasia (BDDH). Preoperative assessment **(a–d)**: **(a)** Anteroposterior pelvic radiograph shows lateral center-edge angle (LCEA) 20.5°, consistent with BDDH. **(b)** Dunn view radiograph reveals alpha angle 55°, indicating mild cam morphology. **(c)** CT scan demonstrates type II anterior inferior iliac spine (AIIS) impingement. **(d)** MRI shows a paralabral cyst with anterior labral base avulsion. Intraoperative arthroscopic procedure **(e–i)**: **(e)** T-shaped capsulotomy using radiofrequency ablation device. **(f)** Arthroscopic shaver debrides redundant inferior extracapsular tissue to prepare for capsular plication. **(g)** Medial capsular closure with interrupted sutures. **(h)** Lateral capsular reconstruction using V-shaped plication technique. **(i)** Arthroscopic view confirming watertight capsular closure. Postoperative follow-up **(j–l)**: **(j)** CT at 24 h shows satisfactory AIIS contouring. **(k)** Immediate MRI demonstrates anatomical capsular coaptation. **(l)** 6-month MRI confirms complete capsular healing without redundancy.

Finally, suturing was performed using a dual-cannula technique through the PMAP and MAP portals. First, the medial points B and B', along with the adjacent medial joint capsule, were approximated using a modified shoelace stitching technique to form an “X” pattern. Second, the central portion (points A and A') was similarly sutured. Finally, the lateral capsule was sutured in the same “X” pattern. This combined approach effectively achieved tightening and elevation of the entire joint capsule (5). The surgical steps are illustrated in Figure 3. A representative case is chronologically illustrated in Figure 4, which is organized into preoperative, intraoperative, and postoperative panels to facilitate interpretation.

#### 2.3.5 Pearls for capsular mobilization and fixation

Given the limited inherent mobility of the hip capsule, effective plication requires careful surgical handling. The key is to adequately mobilize the capsular flaps using a combination of blunt dissection with the arthroscopic shaver and precise grasping with a tissue grasper. Traction should be applied systematically: first, the inferior capsular flap is mobilized superiorly and medially toward the acetabular rim; second, the lateral flap is advanced inferomedially. The grasper should hold the capsule at its robust, fibrous portion to avoid tearing. Sutures are then placed under direct visualization while maintaining traction, ensuring the capsule is secured under appropriate tension to the intended site on the native capsular margin or adjacent soft tissue, achieving a stable, watertight closure.

### 2.4 Postoperative evaluation and follow-up

All patients underwent clinical evaluations preoperatively and at scheduled follow-up visits (postoperative 3, 6, 12 months, and final follow-up). Assessments included the visual analog scale (VAS) for pain (0–10), Modified Harris Hip Score (mHHS, 0–100, with 90–100 excellent, 80–89 good, 70–79 moderate, and <70 poor), Hip Outcome Score-Activities of Daily Living (HOS-ADL), and Hip Outcome Score Sport-Specific Subscale (HOS-SSS).

Rehabilitation protocol: Patients followed a standardized protocol: toe-touch weight-bearing for 4 weeks, passive range of motion initiated immediately, progressive strengthening after 6 weeks, and return to sport after 4–6 months.

### 2.5 Statistical analysis

Statistical analyses were performed using SPSS (version 26.0; IBM Corp., Armonk, NY, USA). The normality of data distribution was verified using the Shapiro–Wilk test. Measurement data conforming to normal distribution are expressed as the mean ± standard deviation. Paired sample t-tests were used to compare preoperative and final follow-up scores. For comparison across multiple postoperative time points within the group, repeated measures analysis was performed with *post-hoc* pairwise comparisons adjusted by Bonferroni correction. *P*-values < 0.05 were considered significant.

## 3 Results

All 15 patients were available for follow-up (minimum 12 months, mean 18.2 ± 4.1 months). The mean preoperative scores were: VAS 5.73 ± 0.57, mHHS 57.33 ± 5.34, HOS-SSS 41.66 ± 5.07, HOS-ADL 50.09 ± 5.02. Significant improvements in all scores were observed at the 3-month postoperative visit (*P* < 0.01 for all compared to preoperative) and were maintained or further improved at subsequent follow-ups. Table 2 presents the detailed scores at each follow-up interval.

**Table 2:** Clinical outcomes at follow-up (mean ± SD).

Time Point	VAS	mHHS	HOS-SSS	HOS-ADL
Pre-op	5.73 ± 0.57	57.33 ± 5.34	41.66 ± 5.07	50.09 ± 5.02
3-month	2.33 ± 0.78*	78.25 ± 4.12*	72.45 ± 5.23*	80.11 ± 3.89*
6-month	1.42 ± 0.67*†	83.17 ± 3.45*†	80.08 ± 4.87*†	86.54 ± 3.01*†
12-month	1.08 ± 0.58*†	85.92 ± 2.89*†	85.33 ± 4.70*†	89.25 ± 2.45*†
Final F/U	1.07 ± 0.57*†	86.07 ± 2.67*†	85.92 ± 4.70*†	89.31 ± 2.43*†

*P < 0.01 vs. Pre-op; †*P* < 0.05 vs. preceding postoperative time point (3-month for 6-month comparison, etc.). Final F/U mean 18.2 months.

## 4 Discussion

When dealing with a dysplastic hip, maintaining acetabular volume is crucial. Acetabular retroversion, which may be a contributing factor to pincer-type FAI, may be indicated by a crossover sign. A surgeon may perform an unnecessary rim excision if they mistakenly interpret a crossover sign as acetabular retroversion. Zaltz et al. (6) found a 50% false-positive rate for the crossover sign in patients with symptomatic FAI and variable AIIS morphology. Radiographic criteria such as an increased Tonnis angle (>10°), increased femoral anteversion, a decreased anterior center-edge angle (ACEA <20°), and a decreased lateral center-edge angle (LCEA <25°) are important indicators of hip dysplasia (7). Acetabular morphology, hip instability, and the severity of the DDH spectrum may impact therapeutic outcomes. Furthermore, the etiology of joint instability involves both soft tissue and bone components. Therefore, the assessment of hip instability and the LCE angle should be part of the diagnosis of BDDH (8, 9).

The treatment of patients with BDDH remains controversial. Current surgical strategies include joint-preserving surgery (e.g., periacetabular osteotomy) and arthroscopic surgery for labral repair. Hip arthroscopy is minimally invasive, making it an attractive option for borderline hip dysplasia. However, rim resection in an already structurally compromised acetabulum can cause iatrogenic hip instability (10, 11). This risk can be mitigated by meticulous management of the capsulolabral structures, focusing on labral repair, precise capsular closure, and preservation of acetabular volume by minimizing rim resection (8, 9). According to a recent comprehensive study by Kraeutler et al. (12), patients with borderline dysplasia can still benefit from hip arthroscopy as a first surgical procedure. However, the authors noted that the studies primarily reported short-term outcomes. Risk factors for arthroscopic treatment failure included a broken Shenton's line, severe femoral anteversion, ligamentum teres tears, age > 42 years, Tonnis grade 1, grade 4 chondral lesions, Tonnis angle > 15°, and vertical center anterior angle < 17°.

Issues surrounding capsular management, including capsulotomy techniques and the extent of closure, have been raised (13). The most commonly used capsulotomy techniques are interportal and T-shaped capsulotomies (14). Capsular management options include complete closure, capsular plication, partial closure, or leaving the capsulotomy unrepaired (15). The hip joint capsule consists of four main ligaments, with the iliofemoral ligament being the strongest and considered a primary stabilizer (16). The use of T-capsulotomy has increased, with a recent systematic review showing it was used in 40% of included studies (17). The T-capsulotomy may extend through the zona orbicularis, a circumferential intracapsular ring that resists distractive forces and functions as an important stabilizer (18). A growing body of biomechanical evidence supports the role of the joint capsule in hip stability (19, 20). The T-capsulotomy improves visualization at the expense of greater capsular disruption. Therefore, careful attention to capsular closure technique is crucial for reducing postoperative instability risk.

There is no consensus on best practices for capsular management after hip arthroscopy. A systematic review shows most studies recommended capsular plication in patients with BDDH, often using the suture shuttle technique described by Domb et al. (21, 22). Capsule closure has become more common recently, partly due to increased awareness of hip microinstability (23, 24). A trend supports capsule closure for improved outcomes compared to no closure. However, a 2020 review by Lin et al. (25) concluded that insufficient data existed to determine if routine capsular repair yields superior clinical results.

Biomechanical studies have shown that capsulotomy increases joint mobility compared to an intact capsule, and that mobility can be restored by repair (26, 27). The most common closure method in current practice is a side-to-side repair using high-strength #2 suture. Robust suture configurations have been shown to enhance hip stability compared to simple sutures.

The arthroscopic T-capsulotomy combined with V-shaped capsular plication technique presented here offers several advantages: (1) enhanced exposure for cam deformity management: the T-shaped capsular incision provides excellent intraoperative exposure of the cam deformity at the femoral head-neck junction, enabling precise decompression; (2) V-shaped capsular plication: this technique is inspired by the Salter-type capsulorrhaphy. It arthroscopically tightens the capsule from superior to inferior and lateral to medial, theoretically compressing the femoral head inferomedially to address deficient acetabular coverage in BDDH; (3) modified shoelace suturing technique: the suturing method is a refined version of a “shoelace suture” technique previously developed by our team, simplifying surgical handling while achieving robust capsular closure and high suture integrity, which biomechanical studies have associated with restored joint stability (26, 27).

Arthroscopy in BDDH remains controversial, with some studies reporting higher failure rates and conversion to total hip arthroplasty. Risk factors for failure, including age > 42 years, Tonnis grade ≥1, and ligamentum teres tears, are only partially addressed in this small cohort. Comparison with contemporary capsular plication series is limited by heterogeneity in technique and outcome measures. Potential risk of over-constraint following aggressive capsular plication should be considered, though no patient exhibited clinical signs of restricted range of motion in this series.

### 4.1 Limitations

This study is limited by its retrospective design, small sample size, absence of a control group, and short follow-up duration. The lack of comparative groups (e.g., alternative capsular management techniques or periacetabular osteotomy) precludes definitive conclusions regarding the superiority of this technique. Important instability-related variables, including femoral anteversion and generalized ligamentous laxity (Beighton score), have been retrospectively supplemented in Table 1. The ACEA was not available due to the absence of standardized false-profile radiographs in this retrospective series. Additionally, the absence of a validated microinstability scoring system limits the objective characterization of instability in this cohort. Concurrent procedures, including variable acetabuloplasty, may contribute to the observed outcomes, and the specific effect of capsular plication alone cannot be isolated. Future prospective, comparative studies with longer follow-up are necessary.

## 5 Conclusions

In conclusion, the surgical technique outlined in this study appears particularly suitable for patients with BDDH or those with significant risk factors for hip joint instability. Short-term outcomes are encouraging, but further studies are required to confirm long-term efficacy and safety.

## Data Availability

The original contributions presented in the study are included in the article/Supplementary Material, further inquiries can be directed to the corresponding author.
